# Harnessing hybrid stacking ensemble learning for accurate pulmonary embolism diagnosis using tabular clinical data

**DOI:** 10.1038/s41598-026-49331-3

**Published:** 2026-05-13

**Authors:** Abeer Abdelhamid, Hossam El-Din Moustafa, Hala B. Nafea, Ehab H. Abdelhay, Mohammed M. Abo-Zahhad, Amir El-Ghamry

**Affiliations:** 1 Faculty of Engineering, Electronics and Communications Engineering Department, Mansoura University, Mansoura, 35516 Egypt; 2 Department of Medical devices, Higher Technological Institute of Applied Health Sciences, Mansoura, Egypt; 3 Faculty of Engineering, Electrical Engineering Department, Sohag University, Sohag, 82524 Egypt; 4 Faculty of Computers and Information, Computer Science Department, Mansoura University, Mansoura, 35516 Egypt; 5 Faculty of Artificial Intelligence and Information, Horus University, 34517, New Damietta, Egypt; 6 Faculty of Engineering, Mansoura National University, Gamasa, Egypt

**Keywords:** Deep Learning (DL), Hybrid Stacking Ensemble (HSE), Marine Predators Algorithm (MPA), Pulmonary Embolism (PE), SAINT (Self-Attention and INtersample Attention Transformer), Computational biology and bioinformatics, Health care, Mathematics and computing, Medical research

## Abstract

Pulmonary Embolism (PE) is a serious condition that can be life-threatening if not diagnosed promptly as timely clinical decisions are critical. This research aims to present a hybrid stacking ensemble (HSE) framework for PE prediction using tabular clinical data from the RSNA-STR-PE dataset. The proposed ensemble combines four complementary base learners: (SAINT transformer, XGBoost, LightGBM, and MLP) to capture diverse feature representations and decision patterns. To optimize ensemble performance, the probabilistic outputs of the base models are weighted using the Marine Predators Algorithm (MPA), enabling adaptive learning of optimal combination weights. These optimized predictions are subsequently fused through a logistic regression meta-learner with L2 regularization, enhancing generalization while mitigating overfitting. Experimental results demonstrate that the proposed MPA-optimized-HSE achieves an accuracy of 92.3% and 0.91 for AUROC, outperforming all individual base models. Furthermore, the MPA-optimized-HSE provides a robust and interpretable approach for improving PE prediction from tabular clinical data, highlighting its potential utility in real-world diagnostic settings.

## Introduction

Pulmonary embolism (PE) is a severe and potentially fatal cardiovascular condition characterized by the obstruction of pulmonary arteries by thrombotic material^1^. In the United States, PE is responsible for approximately 60,000-100,000 deaths annually^2^. It is important to note that about 34% of deaths associated with PE happen abruptly, often before diagnosis or the start of suitable treatment, highlighting the urgent requirement for early detection and intervention^3^. In cases where PE remains undiagnosed, mortality can reach up to 30%, whereas timely diagnosis and treatment can reduce this rate to around 8%^4^. The pathophysiological consequences of PE include impaired pulmonary blood flow, elevated pulmonary arterial pressure, and reduced oxygenation which may ultimately lead to right ventricular dysfunction and hemodynamic instability^5^. In clinical terms, PE represents the third most common acute cardiovascular emergency after myocardial infarction and stroke^6^. Its presentation is highly heterogeneous, spanning from asymptomatic or mild cases conditions that can rapidly progress to shock or cardiac arrest. Frequently observed symptoms include acute shortness of breath (dyspnea), pleuritic chest pain, tachycardia, and indications of right ventricular strain. Severe complications such as ischemia and right-sided heart failure can considerably deteriorate patient prognosis^7^. PE can be classified according to anatomical location (e.g., right-sided, left-sided, or central) as well as temporal progression including acute and chronic forms^8^.

Computerized tomography (CT) and CT pulmonary angiography (CTPA)^9^ are currently regarded as the gold standard imaging modalities for PE diagnosis. However, their interpretation requires specialized expertise and increasing workload on radiologists, coupled with limited availability of trained professionals, poses significant challenges for timely assessment. Additionally, current diagnostic techniques exhibit limitations in detecting segmental and subsegmental emboli and are subject to inter-observer variability, motivating the exploration of alternative and supportive computational approaches^10^. Recent advancements in AI have positioned deep learning (DL)-based image analysis algorithms at the forefront of radiology investigation^11^. In recent decades, numerous methods have been investigated, including stochastic image processing techniques and probabilistic or statistical models utilizing machine learning (ML) algorithms^12^. To overcome these limitations of traditional diagnostic techniques, recent studies have investigated the application of ML and DL methods for predicting PE using structured clinical tabular data which can leverage demographic, vital sign data^13^ and other patient specific features^14^. Such data-driven approaches may uncover subtle patterns indicative of PE, potentially enabling earlier and more personalized risk assessment. However, conventional ML models often struggle to capture complex interactions among heterogeneous features and their predictive performance can be limited when applied to high-dimensional clinical datasets^15^. Ensemble methods, which combine multiple models, have demonstrated enhanced predictive performance and robustness in similar clinical applications^16^. These challenges highlight the need for advanced ensemble strategies capable of effectively combining complementary modeling approaches, improving predictive accuracy, and enhancing modeling generalization on complex tabular clinical data.

Although the RSNA-STR-PE Challenge dataset has been extensively investigated in image-based DL studies^17,18^, the tabular clinical component of the dataset has received comparatively limited attention. Current studies utilizing the CSV-based clinical data typically rely on single model approaches, which may inadequately capture weakly correlated, heterogeneous clinical features^19^. Moreover, modern transformer-based architectures designed for tabular data such as SAINT (Self-Attention and INtersample Attention Transformer) remain underexplored in medical applications ^20^. To the best of our knowledge, this is the first study to integrate transformer-based attention mechanisms, neural networks, and tree-based models in a metaheuristic optimized hybrid stacking ensemble for tabular clinical PE prediction. Despite advances in DL and ML for PE prediction, there remains a clear need for methods that can effectively integrate heterogeneous tabular clinical features and leverage modern ensemble strategies.

In this study, we propose a metaheuristic-optimized hybrid stacking framework for PE prediction using structured clinical metadata. Although the study does not introduce a new standalone learning algorithm, its contribution lies in the systematic integration and adaptive optimization of heterogeneous models specifically tailored for PE classification.

The main contributions of this research are summarized as follows:

- Development of a unified hybrid stacking ensemble that integrates SAINT transformer, XGBoost, LightGBM, and MLP to capture complementary nonlinear and contextual relationships within tabular clinical data.
- Introduction of a joint optimization strategy using the Marine Predators Algorithm (MPA) to simultaneously optimize base model hyperparameters and ensemble weights within a unified search space.
- Comprehensive experimental evaluation including ablation studies, comparison of multiple ensemble fusion strategies, and explainability analysis using SHAP and permutation feature importance.
- Benchmarking on the publicly available RSNA-STR-PE dataset to ensure reproducibility and standardized comparison with future studies.
- Demonstration of consistent performance improvement over individual learners and conventional ensemble strategies.

The structure of the rest of this paper is organized as follows: Section ”Related work” provides an overview of related studies, highlighting recent advancements and existing limitations in automated PE detection using ML/AI methods. The section ”Materials and methods” depicts the detailed methodology of the proposed methodology, outlining the preprocessing techniques, the hybrid stacking ensemble framework, and the specifics of our experimental setup. The section ”Experimental results” presents the experimental results, including comprehensive performance evaluation metrics and comparative analysis with state-of-the-art methods. The section ”Discussion” provides a discussion on the implications, strengths, limitations, and practical considerations of our proposed method. Finally, the section ”Conclusion and future directions” concludes the paper and suggests potential directions for future research.

## Related work

PE is a potentially fatal condition and precise prediction is essential for timely clinical intervention, motivating the application of artificial intelligence (AI) and ML approaches to support early detection. Recent survey studies^21^ have highlighted the growing impact of AI in PE diagnosis using CTPA images. These works emphasize the importance of diverse datasets, advanced architectures, and multimodal learning strategies, including weak supervision, contrastive learning, and image quality enhancement. They also report that publicly available datasets such as RSNA-STR-PE have been widely used to develop high-performance models, and that integrating multiple sources of information can improve robustness and generalization of PE detection systems. Recent advances in ML and AI have significantly influenced the prediction and diagnosis of PE, providing alternatives or complementary approaches to traditional clinical risk scores such as the Wells and Geneva scores. Various ML models including Random Forest (RF), XGBoost (XGB), Support Vector Machines (SVM) models, and neural networks have demonstrated strong predictive performance across diverse patient populations. Ensemble ML approaches have also been widely applied for cardiovascular disease prediction, demonstrating the effectiveness of combining multiple classifiers such as XGBoost, AdaBoost, and Random Subspace with feature selection techniques, achieving high predictive performance with reported accuracy around 96%. Systematic reviews and meta analysis reported AUROC values around 91%-93% for mortality prediction, consistently outperforming conventional scoring systems^22^.

Several studies have explored ML models on structured clinical tabular to improve risk stratification across various patient cohorts using ML models like (XGB, LightGBM (LGBM), and RF) achieved accuracies between (85%-89%) and high sensitivity while reducing unnecessary imaging^14^. Similarly, early PE detection using routine clinical and laboratory variables with XGB yielded AUROC of 0.87, showing potential for rapid pre-screening prior to imaging^23^. Explainable ML approaches have also been employed, revealing the importance of features such as D-dimer levels, RV/LV ratio, and patient age with SHAP analysis highlighting their influence on risk prediction^24^. Beyond tabular data, non-imaging modalities such as 12-lead ECG signals have been used for PE prediction, achieving AUROC values around 0.85 and outperforming conventional clinical scores^25^. Also, recent ECG-based DL approaches combining multimodal transformations and CNN models^26^ have achieved high classification performance, with reported accuracy exceeding 99% along with similarly high precision, recall, and F1-score. In addition, Hybrid DL approach has also been applied by Saranya et al.^27^ to photoplethysmography (PPG) signals for arrhythmia classification, combining CNNs with attention-based recurrent architectures (BiLSTM model) to capture both spatial and temporal features, achieving competitive performance with accuracy around 89%. Imaging based-approaches using CTPA scans with 3D CNNs (e.g., 3D ResNet) combined with XGB have demonstrated accuracy up to 94.5%, although studies are often limited by small sample sizes and potential overfitting^28^. Recent studies have also explored transformer-CNN hybrid architectures for PE segmentation in CTPA images. For example, a hybrid SwinUNet combined with ResNet-152 and optimized using metaheuristic techniques proposed by Mulam et al.^29^ demonstrated very high segmentation performance on the RSNA-STR-PE dataset, highlighting the effectiveness of combining transformer-based attention mechanisms with CNN feature extraction for fine-grained image segmentation. In addition to segmentation focused approaches, recent studies have proposed AI-based triaging models for PE detection from CTPA scans. For instance, an attention-based DL framework demonstrated by Singh et al.^30^ improved diagnostic performance compared to conventional CNN and CNN-LSTM models, achieving an AUC of 0.95 and significantly reducing inference time, highlighting the potential of AI-assisted workflows to accelerate clinical decision-making. Multimodal fusion models that integrate tabular clinical data, imaging, and laboratory tests have further improved predictive performance, achieving AUROC values between (0.92-0.94)^31^.

Recently, transformer-based architectures have emerged as powerful tools for modeling tabular clinical data. Models such as SAINT are capable of capturing dependencies within individual samples as well as patterns across samples, enabling higher order feature interactions without the need for manual feature engineering. Benchmark studies show that SAINT consistently matches or outperforms traditional gradient-boosted decision trees such as XGB and LGBM, and ensembles combining SAINT with tree-based or neural network models provide additional gains in performance and stability^28^. In addition, vision transformers (ViT) combined with CNNs have also achieved near perfect slice-level PE classification on the RSNA-STR-PE dataset with AUROC around 0.99^8^.

Despite these advances in ensemble and hybrid ML models, few studies applied metaheuristic optimization techniques to enhance ensemble performance in clinical predictive tasks. Recent studies have also explored metaheuristic optimization techniques for cardiovascular disease prediction^32^, where feature selection and model training are enhanced using algorithms such as Binary Krill Herd optimization combined with DL models, achieving high predictive performance with accuracy around 95%. These findings highlight the potential of metaheuristic optimization strategies in improving predictive modeling performance. Metaheuristic algorithms such as MPA have been developed for global search and hyperparameter optimization in ML models, improving performance by efficiently exploring complex parameter spaces^33^. Recent studies also highlight the growing interest in metaheuristic-based ensemble learning for selective ensemble design and optimization across various application domains, though their use in biomedical prediction remains limited^34^. To the best of our knowledge and based on the available literature, no previous study has applied a metaheuristic-optimized hybrid stacking ensemble to publicly available tabular clinical PE datasets, including RSNA-STR-PE. This underscores a clear research gap in reproducible benchmarking and advanced ensemble modeling for structured clinical data, which the present study directly addresses. By leveraging the publicly available RSNA-STR-PE tabular dataset, our work provides the first reproducible evaluation of such an approach for PE prediction.

Table 1 summarizes recent studies on PE prediction and detection using AI models. It illustrates the variability in data modalities, model architectures, and reported performance while highlighting the potential of hybrid and optimization-based approaches for more robust and generalizable PE prediction. Taken together, these studies highlight the progress in AI/ML for PE prediction but also underscore the unexplored potential of metaheuristic algorithms which could integrate diverse models to improve robustness and predictive performance, an opportunity our study addresses.

**Table 1:** Summary of recent studies on PE detection using ML and AI models.

Study	Dataset / Data Type	Method	Sample Size	Performance	Limitations
Lian et al.^35^	EHR (hospital data)	XGB, RF, LR, SVM	312 pts	AUC 0.87	Small sample size, single-center
Zhou et al.^24^	Tabular clinical	RF, others	1,480 pts	AUC 0.77 for RF	Moderate validation, EHR coding.
Mesinovic et al.^36^	Tabular clinical	Gradient Boosting	800,000+ pts	AUROC 0.76	Lack of imaging data.
Tur et al.^28^	CTPA imaging	3DResNet + XGBoost	193 scans	Accuracy 94.0%	Small dataset; risk of overfitting
Ferreira et al.^25^	ECG (signal data)	Ensemble DL	1,014 ECGs	AUC 0.75	Limited to moderate cases.
Wang et al.^37^	MIMIC-IV (tabular EHR)	XGB, LR	1,229 pts	AUC 0.82 for XGB	ICU-only, may not generalize
Eini et al.^22^	Tabular clinical (EHR)	ML models	17 studies; N = 844,071	AUROC 0.91	Heterogeneity, retrospective
Ma et al.^38^	Tabular clinical	ML models (RF, XGBoost, SVM, NN)	Multiple datasets (N= 596,092 pooled)	Pooled sensitivity 79.0%	Limited external validation
Kong et al.^14^	Tabular clinical + lab data	RF, XGB, LGBM	2,350 patients	RF achieved best generalization; AUC= 0.90	Dataset specific, may not generalize
Kim et al.^39^	Tabular clinical + demographics	RF	585 pts	Decreased unnecessary CT scans AUROC 0.73	Single-center data, small sample
Sezer et al.^40^	Tabular clinical + echo + ECG	MLP	1,580 pts	specificity = 89%	Limited validation, interpretability
Cahan et al.^31^	CTPA + EHR	multimodal fusion (Bilinear attention + TabNet)	450 pts	sensitivity 90%	Small datasets, short-term prediction
Mulam et al.^29^	CTPA imaging (RSNA-STR-PE)	Transformer-CNN hybrid (SwinUNet + ResNet-152) with optimization	RSNA-STR-PE dataset	Accuracy = 99.95%, Specificity = 99.12%	limited generalization beyond dataset
Singh et al.^30^	CTPA imaging (RSNA-STR + in-house + external datasets)	Attention-based DL (CNN + attention; comparison with CNN and CNN-LSTM)	7279 (RSNA-STR-PE), 106 (external), 200 (efficiency dataset)	AUC = 0.95, Accuracy = 88%	Performance depends on dataset distribution; limited interpretability
Tiwari et al.^21^	Structured/tabular clinical data	Ensemble of multiple ML classifiers (e.g., RF, XGB, SVM)	Not explicitly specified (varies across datasets)	Not explicitly specified (varies across datasets)	Dataset-dependent; lacks standardized benchmarking; no metaheuristic optimization or transformer-based models

## Materials and methods

**Fig. 1:**
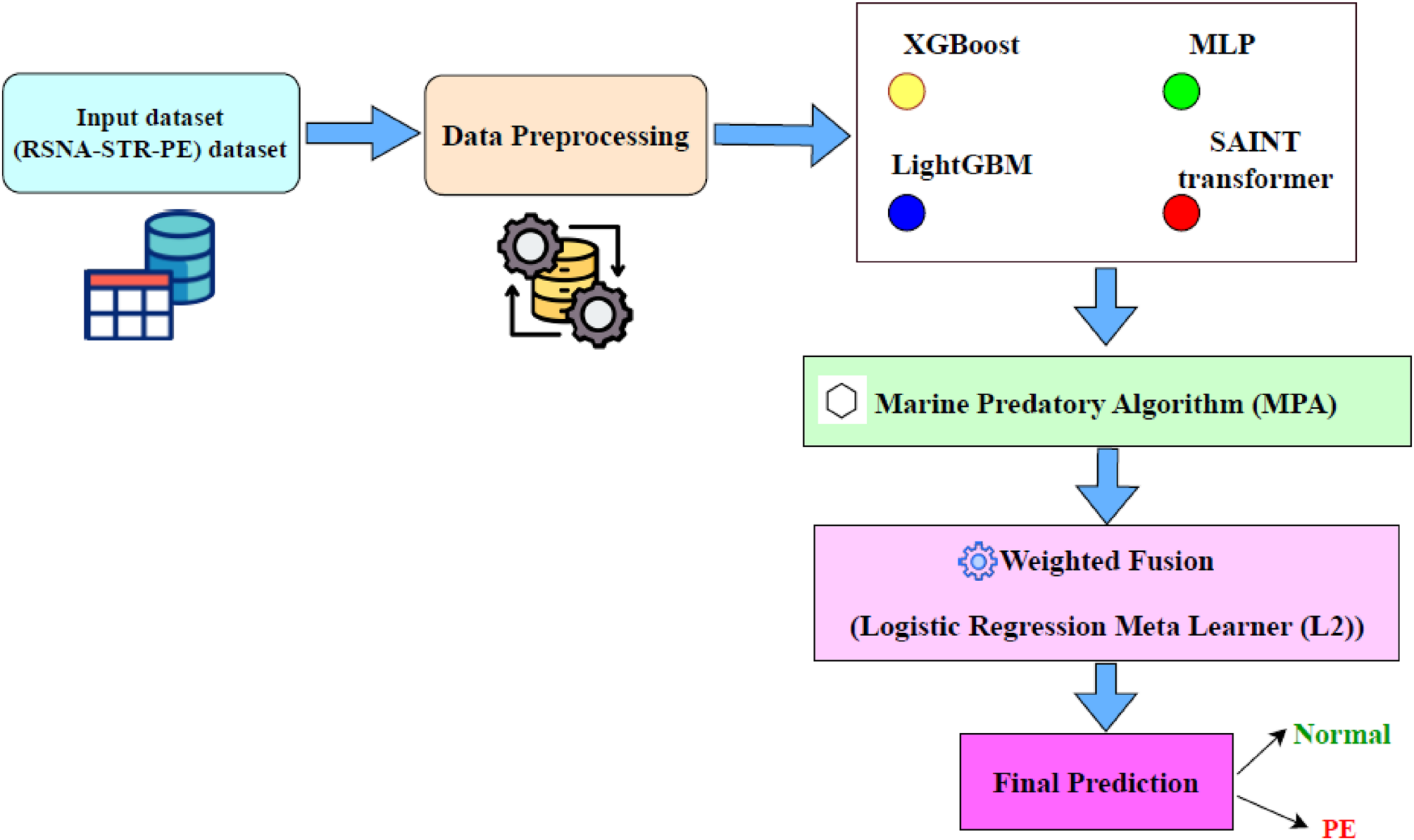
Blockdiagram of the proposed MPA-optimized-HSE technique.

The proposed framework consists of a multi-stage pipeline as illustrated in Fig. 1. The workflow begins with data preprocessing of the RSNA-STR-PE tabular metadata, followed by training multiple heterogeneous base learners including SAINT transformer, XGBoost, LightGBM, and MLP. The probabilistic outputs of these models are then combined using a hybrid stacking ensemble, where a logistic regression meta-learner integrates the predictions. Furthermore, the Marine Predators Algorithm (MPA) is employed to jointly optimize the base model hyperparameters and ensemble weights to enhance overall predictive performance.

### Dataset

The dataset utilized in this study was obtained from RSNA-STR-PE detection challenge^41^, which provides CTPA exams annotated for presence of PE. Each record in the accompanying metadata file corresponds to an individual CT image slice (DICOM format) and is identified by three-level hierarchical structure:

1. StudyInstanceUID: a unique identifier for each CTPA examination (patient-level study).
2. SeriersInstanceUID: a unique identifier assigned to each image series within the study.
3. SOPInstanceUID: a unique identifier for each image slice.

The dataset contains both image-level and exam-level diagnostic annotations, as well as quality assurance indicators provided by expert thoracic radiologists including:

1. pe-present-on-image: image-level label indicating whether any PE is visible.
2. negative-exam-for-pe: exam-level label indicating PE absence.
3. qa-motion, qa-contrast, and flow-artifact: informational flags recorded by radiologists to describe issues related to patient motion, contrast quality, or flow artifacts, respectively.
4. Right-to-left-ventricular (RV/LV) ratio indicators: (rv-lv-ratio-gte-1 and rv-lv-ratio-lt-1).
5. Anatomical location labels: leftsided-pe, rightsided-pe, central-pe
6. Chronicity labels: chronic-pe and acute-and-chronic-pe.
7. true-filling-defect-not-pe defect and indeterminate.

The dataset includes thousands of CTPA studies totaling over 700,000 image slices, each annotated and reviewed by expert thoracic radiologists. These annotations enable both image-level and study-level modeling of PE presence and characteristics. In this work, only structured tabular metadata was utilized as input for the proposed model, enabling PE classification without direct use of imaging data.

### Data preprocessing

Prior to model training, several preprocessing steps were applied to ensure data consistency and prevent information leakage^42^. Non-informative identifiers (StudyInstanceUID, SeriesInstanceUID, and SOPInstanceUID) were retained exclusively for grouping and cross-validation purposes and excluded from the model feature space. Missing numerical values were imputed using the median according to eq 1, while categorical and binary features were imputed using the mode according to eq 2. Binary variables were encoded as 0 and 1, while multi-class categorical variables were transformed via one-hot encoding^43,44^. Continuous variables were standardized using z-score normalization.

1 $$\begin{aligned} x_i^{\text {imputed}} = \text {median}\big (x_{\text {observed}}\big ) \end{aligned}$$

where $x_i^{\text {imputed}}$ is the imputed value for the missing entry, $x_{\text {observed}}$ are the observed values of the feature, and $\text {median}()$ is the median of these observed values. and for a categorical feature c as:

2 $$\begin{aligned} c_i^{\text {imputed}} = \text {mode}\big (c_{\text {observed}}\big ) \end{aligned}$$

where $c_i^{\text {imputed}}$ is the imputed value for the missing categorical entry, $c_{\text {observed}}$ are the observed values of the categorical feature, and $\text {mode}()$ is the most frequent value among these observed values.

3 $$\begin{aligned} x_i^{\text {standardized}} = \frac{x_i - \mu _x}{\sigma _x} \end{aligned}$$

where, $x_i^{\text {standardized}}$ represents the standardized value, $x_i$ is the original value, $\mu _x$ is the feature mean, and $\sigma _x$ is the feature standard deviation. To address class imbalance, stratified 5-fold cross validation was applied, ensuring that class proportions were maintained across folds. After preprocessing, all features were aggregated into a unified feature matrix^45^

$$\begin{aligned} \textbf{X} \in \mathbb {R}^{N \times F} \end{aligned}$$

4 $$\begin{aligned} \textbf{X} = \begin{bmatrix} x_{11} & x_{12} & \dots & x_{1F} \\ x_{21} & x_{22} & \dots & x_{2F} \\ \vdots & \vdots & \ddots & \vdots \\ x_{N1} & x_{N2} & \dots & x_{NF} \end{bmatrix} \in \mathbb {R}^{N \times F} \end{aligned}$$

where N is the number of samples and F is the number of features.

### Base models

To capture complementary characteristics of the tabular clinical dataset, four base models were employed:

1. **XGB:** a gradient boosted decision tree model capable of handling non-linear feature interactions^46,47^. Hyperparameters were optimized via cross-validation.
2. **LGBM:** a highly efficient for large datasets gradient boosting framework leaf-wise tree growth, providing complementary performance to XGB^48^.
3. **Multilayer Perceptron (MLP):** a fully connected neural network with ReLU activation and dropout regularization, serving as a neural baseline^19^.
4. **SAINT transformer:** a transformer-based model developed for structured tabular datasets, leveraging attention mechanisms to learn dependencies among features^49^.

### SAINT transformer architecture

The SAINT architecture was employed to model both intra-sample (feature-level) and Inter-sample (patient-level) relationships, which are critical for clinical prediction tasks.

#### Input representation

- Numerical features were embedded using linear transformations.
- Categorical features were represented using learnable embedding layers (embedding dimension = 128).
- Positional embeddings were added to preserve feature ordering information^50^.

#### Transformer encoder blocks

The model consists of four stacked encoder blocks, each containing:

a) Multi-head self-attention (8 heads), defined as:

5 $$\begin{aligned} \text {Attention}(Q, K, V) = \text {softmax} \left( \frac{QK^\top }{\sqrt{d_k}} \right) V \end{aligned}$$

where:

- $$Q$$: Query matrix
- $$K$$: Key matrix
- $$V$$: Value matrix
- $d_k$: Dimensionality of the key vectors (used for scaling)
- $\text {softmax}$: Applied row-wise to normalize attention scores

6 $$\begin{aligned} \text {MultiHead}(Q, K, V) = \text {Concat}(\text {head}_1, \dots , \text {head}_h)W^O \end{aligned}$$

7 $$\begin{aligned} \text {head}_i = \text {Attention}(QW_i^Q, KW_i^K, VW_i^V) \end{aligned}$$

where:

- $Q, K, V$ are the query, key, and value matrices.
- $W_i^Q, W_i^K, W_i^V$ denote the learnable projection matrices for the $i^{th}$ attention head.
- The output projection matrix $W^O$ is applied following the concatenation of all attention heads.
- $\text {Attention}(Q, K, V) = \text {softmax}\left( \frac{QK^T}{\sqrt{d_k}} \right) V$ denotes the scaled dot-product attention.

b) Layer normalization^51^ after each sub-layer.

8 $$\begin{aligned} \text {LayerNorm}(x) = \frac{x - \mu }{\sigma + \epsilon } \cdot \gamma + \beta \end{aligned}$$

where:

- $x$ is the input vector.
- $\mu$ is the mean of the elements in $x$.
- $\sigma$ is the standard deviation of the elements in $x$.
- $\epsilon$ is a small constant added for numerical stability.
- $\gamma$, $\beta$ are learnable scaling and shifting parameters.

c) Feed-forward networks (FFN) with RelU activation^52^ activations.

9 $$\begin{aligned} \text {FFN}(x) = \text {ReLU}(xW_1 + b_1)W_2 + b_2 \end{aligned}$$

where:

- $x$ is the input feature vector for a single sample.
- $W_1, W_2$ are learnable weight matrices of the first and second fully connected layers, respectively.
- $b_1, b_2$ are bias vectors added after each linear transformation.
- $\text {ReLU}(\cdot )$ is the Rectified Linear Unit activation function, defined as $\text {ReLU}(z) = \max (0, z)$.

d) Dropout regularization ( rate = 0.1) for regularization.

Intra-sample and inter-sample attention mechanisms were alternated across layers to capture complementary interaction patterns.

#### Output layer

A fully connected layer with sigmoid activation produced the final binary classification (PE present or absent).

### Proposed hybrid stacking ensemble (HSE)

For the proposed HSE model, it was designed to combine probabilistic predictions from all base models. These outputs were used as input features to a logistic regression meta-learner with L2 regularization, enabling optimal weighting of individual base models while mitigating overfitting. Let the prediction vector be defined as:

10 $$\begin{aligned} \textbf{p} = [p_1,\, p_2,\, p_3,\, p_4] \end{aligned}$$

where each $p_i$ denotes the predicted probability from the *i*-th base model. The meta-learner computes the final ensemble predictions as:

11 $$\begin{aligned} \hat{y} = \sigma (\textbf{w}^\top \textbf{p} + b), \end{aligned}$$

where $\sigma$ denotes the sigmoid activation function, $\textbf{w}$ corresponds to the learned weight vector, and *b* indicates the bias term.

The meta-learner determines optimal weights for each base model while penalizing excessively large coefficients, reducing overfitting and enhancing generalization. The training objective is expressed as:

12 $$\begin{aligned} \mathcal {L} = -\frac{1}{N}\sum _{i=1}^{N} \left[ y_i \log (\hat{y}_i) + (1 - y_i)\log (1 - \hat{y}_i) \right] + \lambda \left\Vert \textbf{w} \right\Vert _2^2, \end{aligned}$$

L2 regularization penalizes excessively large coefficients, reducing overfitting and enhancing generalization. Stacking allows the ensemble to combine complementary strengths of tree-based, neural network, and transformer models, improving both accuracy and robustness.

### Metaheuristic optimization for base model hyperparameters and ensemble weights

To further enhance predictive performance, MPA was employed to jointly optimize base model hyperparameters and ensemble weights. It is a nature inspired metaheuristic optimizer based on the hunting strategies of marine predators^53^. This algorithm was used to simultaneously optimize both the hyperparameters of the base models (SAINT, XGB, LGBM, and MLP) and the stacking ensemble weights. Validation accuracy was used as the fitness function, enabling efficient exploration of the high-dimensional optimization space^54^.

The Neural network-based models (MLP, SAINT) were trained using the Adam optimizer^55^, with an initial learning rate of 0.001, binary cross entropy (BCE) loss, and early stopping (patience = 3). Weight decay (1e-5) and cosine learning rate with warm-up were applied. Stratified 5-fold cross-validation was used to preserve the proportion of PE-positive and PE-negative cases in each fold. Class weights were incorporated in the BCE loss function to address class imbalance. BCE can be defined as:

13 $$\begin{aligned} \mathcal {L} = - \frac{1}{N} \sum _{i=1}^{N} \left[ y_i \log (\hat{y}_i) + (1 - y_i) \log (1 - \hat{y}_i) \right] \end{aligned}$$

where $y_i$ is the true label, $\hat{y}_i$ is the predicted probability, and N represents the batch size.

Tree-based models (XGB and LGBM) were trained using optimized gradient boosting configurations. The hyperparameters of the base models were determined using a combination of GridSearchCV, stratified 5-fold CV and empirical tuning with further optimization performed using MPA. Table 2 displays the selected parameters.

**Table 2:** The hyperparameters used for the base models within the proposed MPA-optimized-HSE model.

Base Model	Type	Key Hyperparameters	Values
SAINT Transformer	Neural Network	Optimizer	Adam, lr = 0.001
		Loss Function	BCE with class weights
		Weight Decay	1e-5
		Epochs	50
		Batch Size	32
MLP	Neural Network	Optimizer	Adam, lr = 0.001
		Loss Function	BCE with class weights
		Weight Decay	1e-5
		Epochs	50
		Batch Size	32
XGBoost	Tree-based	n_estimators	200
		max_depth	6
		learning_rate	0.05
		subsample	0.8
		colsample_bytree	0.8
		objective	’binary:logistic’
LightGBM	Tree-based	n_estimators	200
		num_leaves	64
		max_depth	6
		learning_rate	0.05
		feature_fraction	0.8
		bagging_fraction	0.8
		bagging_freq	1
		objective	’binary’

## Evaluation metrics

To assess the performance of the proposed model, we utilize accuracy, recall, specificity, and AUROC as critical metrics. To calculate accuracy, the number of correctly identified samples is divided by the total number of samples. It can be calculated mathematically as expressed in Eq 14 where, tp, tn, fp, and fn denote true positive, true negative, false positive and false negative, respectively^56^. Also, precision refers to the percentage of accurately anticipated positive patterns inside a positive class, while recall measures the percentage of correctly classified positive patterns according to Eqs. (15, 16). Additionally, F1-score^57^ reflects the harmonic mean of precision and recall as depicted from Eq. 17.

14 $$\begin{aligned} \text{ Acc }=\frac{tp+tn}{tn+tp+fn+fp} \end{aligned}$$

15 $$\begin{aligned} \text{ Pre }=\frac{tp}{tp+fp} \end{aligned}$$

16 $$\begin{aligned} \text{ Rec }=\frac{tp}{tp+fn} \end{aligned}$$

17 $$\begin{aligned} F1-score = \frac{2tp}{2tp+fp+fn} \end{aligned}$$

## Experimentation and performance

This section presents the experimental findings of the proposed model on the dataset and compares them to other established models. All experiments were conducted using stratified 5-fold cross-validation. This ensured that the proportions of PE-positive and PE-negative cases were preserved across all folds, thereby preventing class imbalance from affecting model evaluation. Performance metrics—including accuracy, precision, recall, F1-score, and AUROC—were computed for each fold and averaged across the five folds to provide robust estimates of model performance. The reported results in Tables 4, 5, 6)reflect these averaged metrics, demonstrating the effectiveness of the proposed MPA-optimized HSE model. All experiments were performed on an NVIDIA T4 GPU with 16 GB of GPU memory and 12 GB RAM, utilizing Python 3.8 and Tensorflow 2.14.0 framework. Training times are reported per fold under the specified hardware configuration. The total training time for the proposed MPA-optimized-HSE across the 5-fold cross-validation was approximately 3–4 hours. The performance of the models was assessed using accuracy, precision, recall, and F1-score. As summarized in Table 4, the MLP model achieved an accuracy of 79.5% with slightly lower precision and recall values. LGBM yielded an accuracy of 78.3% while XGB achieved an accuracy of 85.7% and F1-score of 81.6%. In addition, SAINT transformer demonstrated an accuracy of 90.4% with balanced precision (87.1%) and recall (85.2%), and F1-score of 86.6%. Conversely, the proposed MPA-optimized-HSE model outperformed all individual base models in all metrics. Specifically, it scored an accuracy of 92.3%, precision of 88.2%, recall of 87.4%, and F1-score of 89.5%, outperforming all individual base models.

**Fig. 2:**
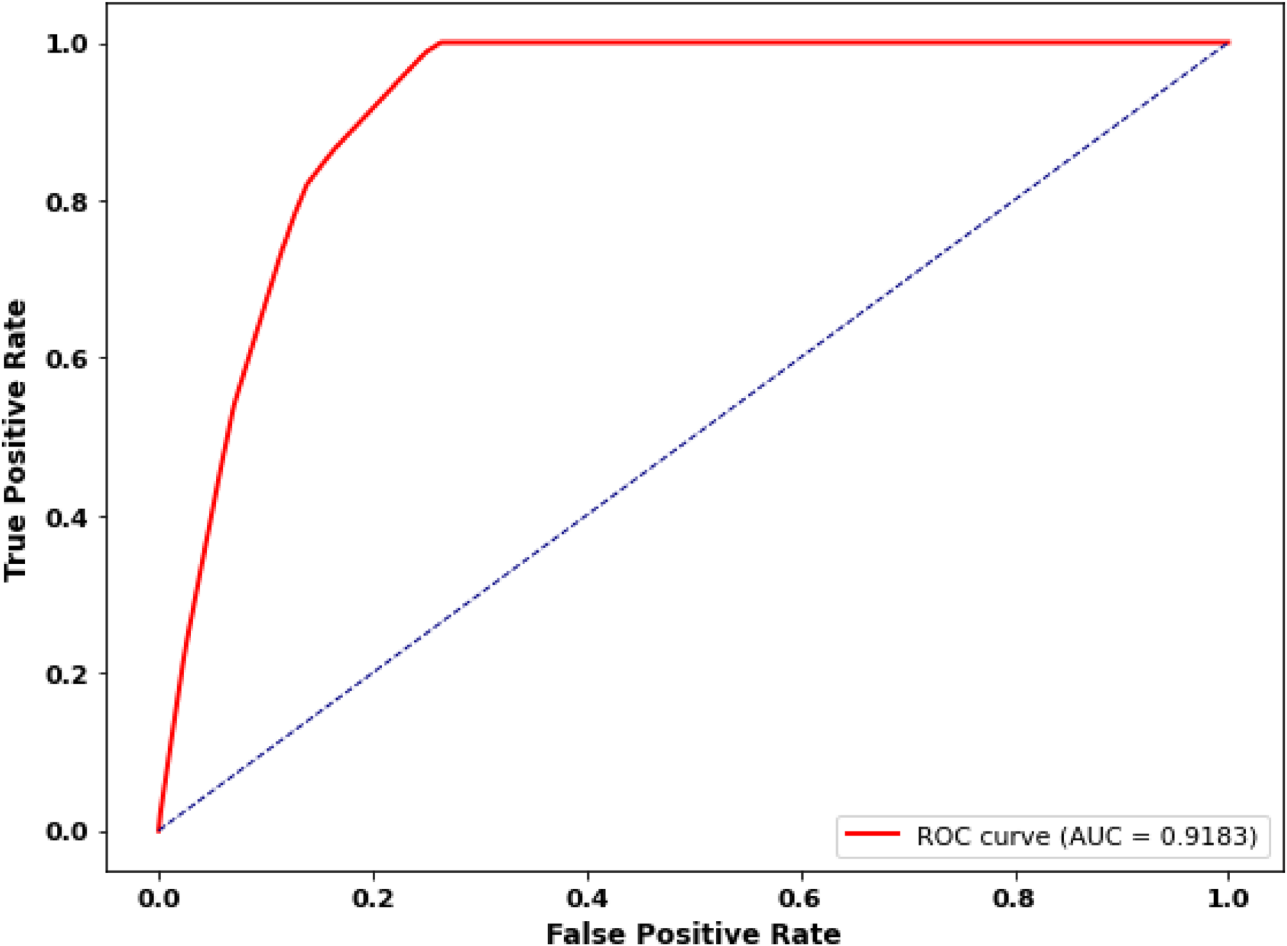
ROC curve for the proposed MPA-optimized-HSE model.

**Fig. 3:**
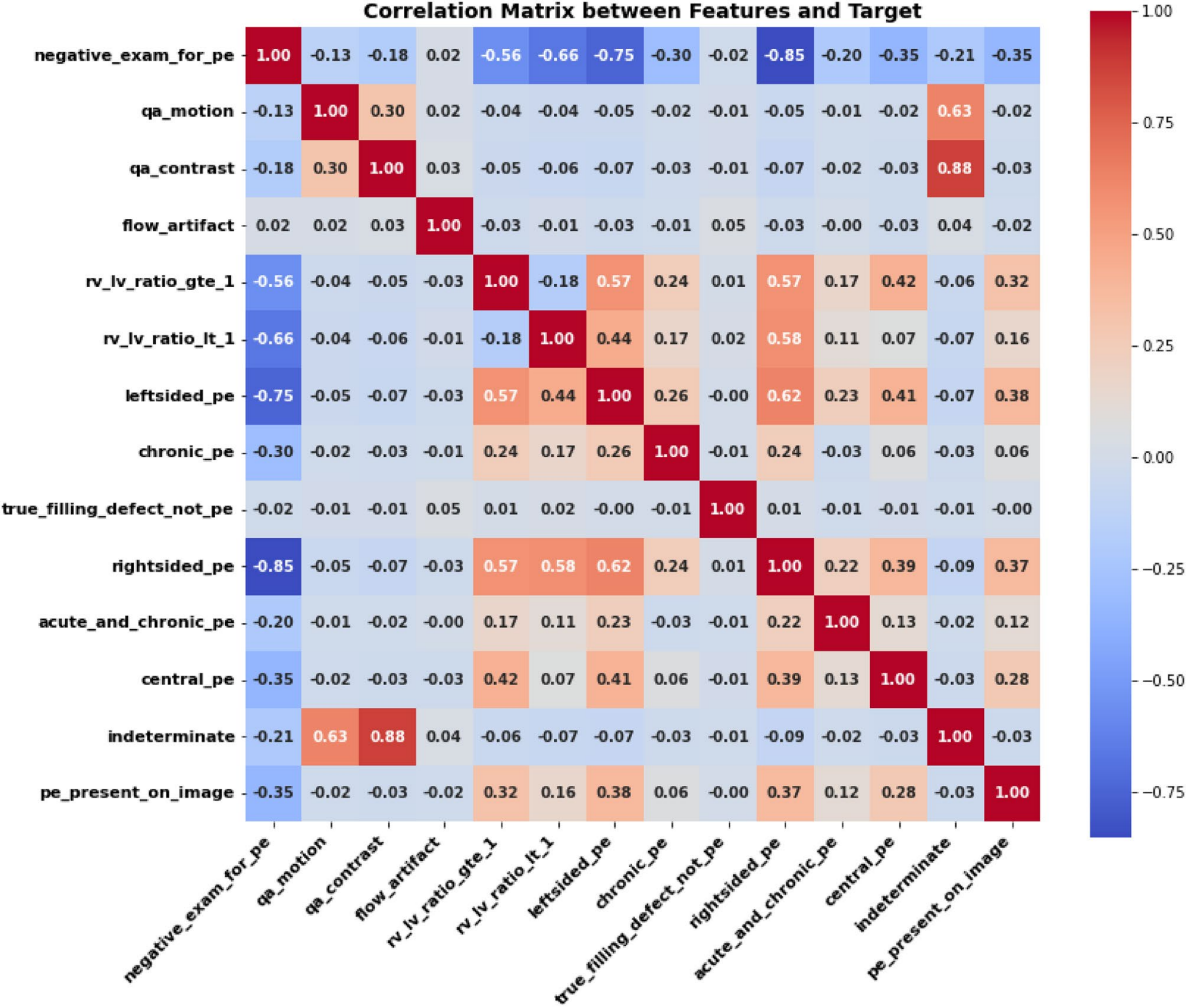
Correlation matrix for the proposed HSE model.

To further evaluate classification performance, ROC curve and the correlation matrix were constructed. As shown in Fig. 2, the ROC curve demonstrates strong discriminative capability with an AUC of 0.91. Also, Fig. 3 illustrates the correlation matrix between the extracted features and the target variable. Strong negative correlations were observed between PE presence and several clinically relevant features, including right-sided PE (r = -0.85), infacted PE ( r = -0.75), and RV/LV ratio indicators (r = (-0.56 and 0.66). Moderate negative correlations were also noted for central PE ( r = -0.35) and chronic PE ( r = -0.30). In contrast, imaging artifact-related features exhibited weak correlations with the target variable, suggesting a limited direct influence. Also, positive correlations were observed between PE subtypes and RV/LV ratio features, reflecting known clinical associations.

### Impact of MPA optimization

To assess the contribution of MPA optimization, the performance of the proposed model was compared before and after optimization. Prior to MPA optimization, the proposed model achieved an accuracy of 90.6%. After applying MPA, accuracy increased to 92.3% accompanied by consistent improvements in precision, recall, and F1-score as obtained in Table 3. These findings demonstrate that MPA optimization enhances the proposed model’s performance by jointly refining base model hyperparameters and ensemble weighting coefficients.

**Table 3:** Impact of MPA optimization on the performance of the proposed HSE model.

Metric	Before MPA (%)	After MPA (%)
Accuracy	90.6	92.3
Precision	87.0	88.2
Recall	85.0	87.4
F1-score	87.8	89.5

### Explainability and feature importance

**Fig. 4:**
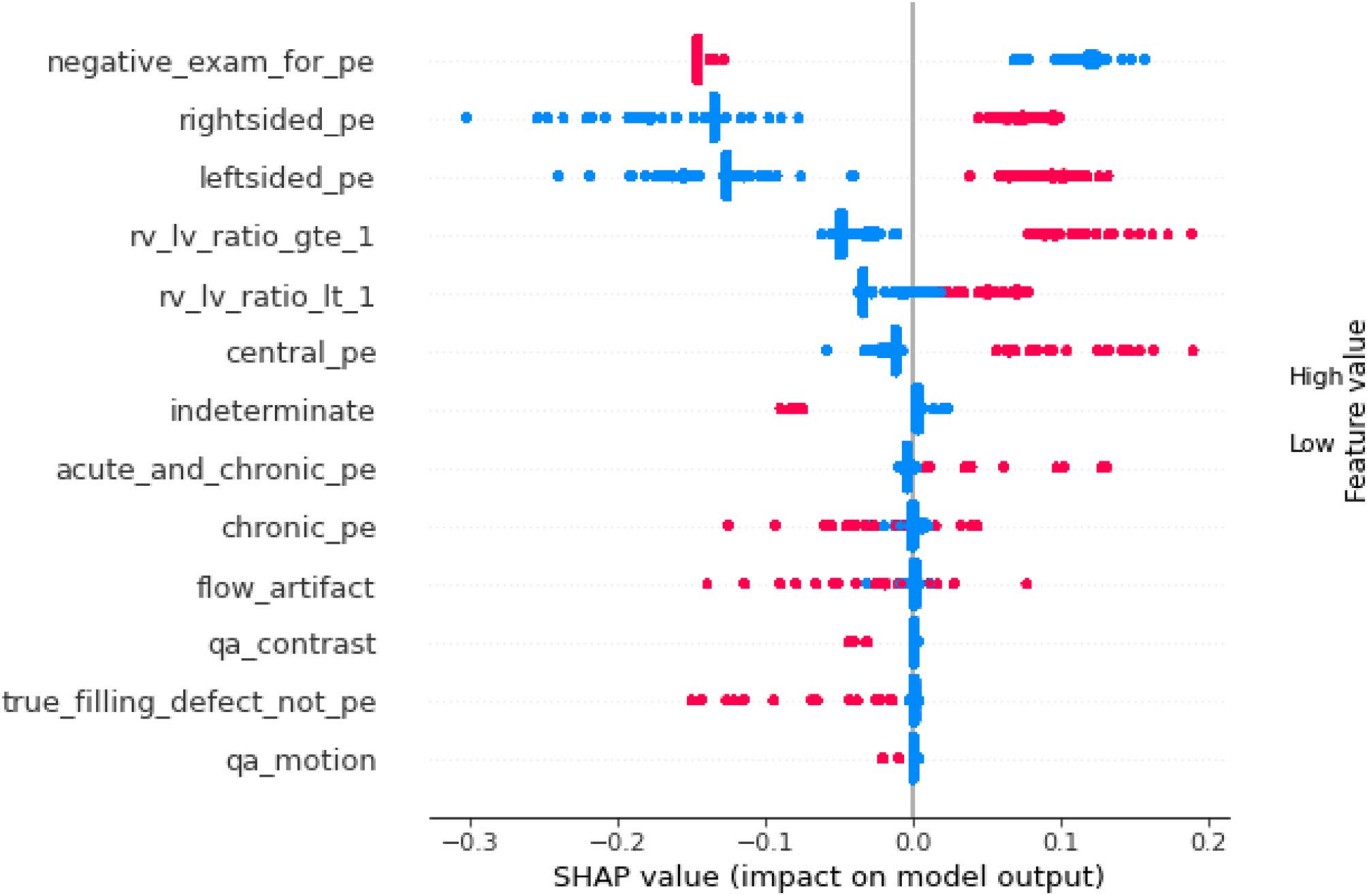
SHAP summary plot showing the most influential features in PE prediction.

**Fig. 5:**
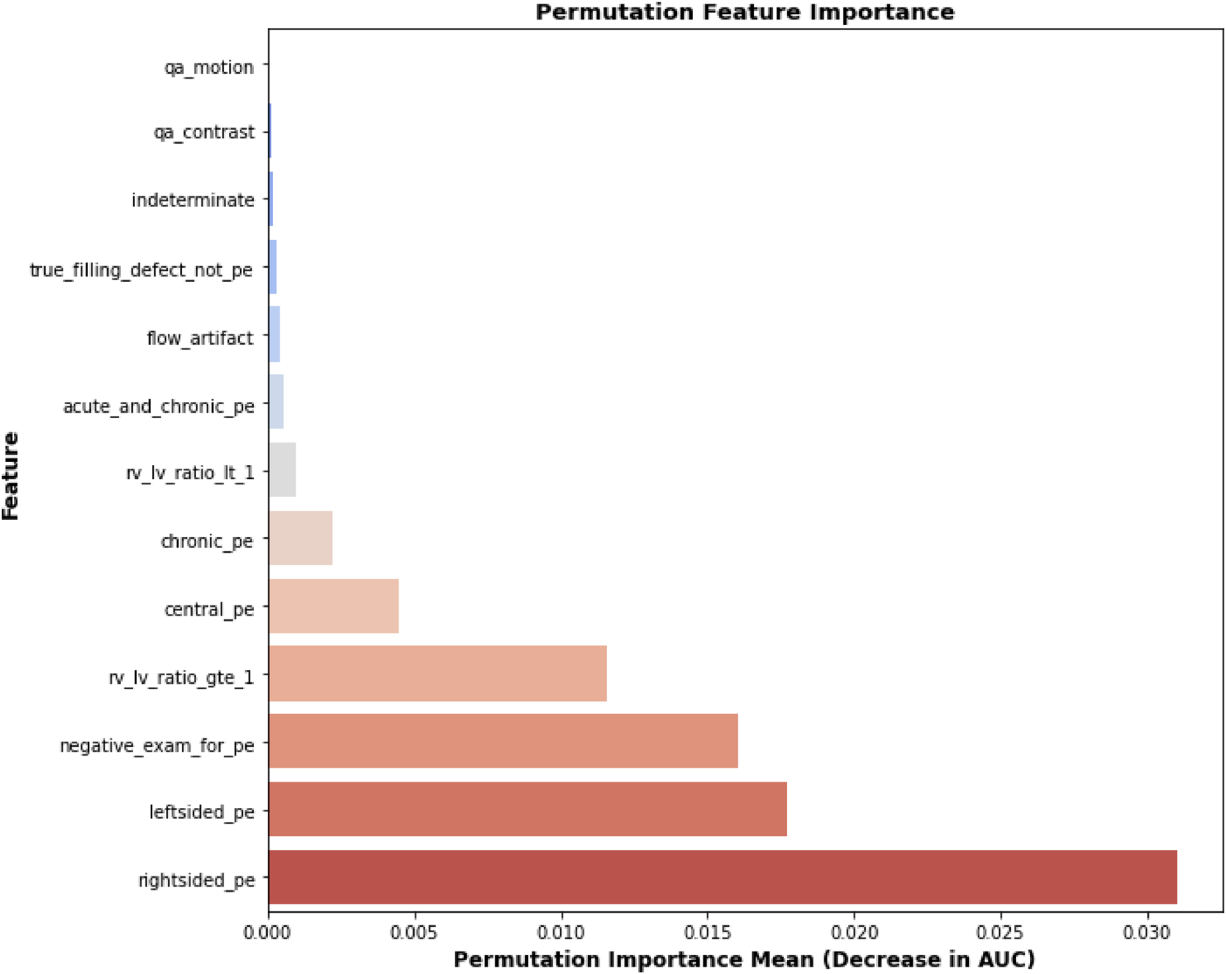
Permutation importance for the proposed HSE model.

To further understand the decision-making behavior of the proposed MPA-optimized-HSE model, model agnostic interpretability analysis was conducted using SHAP and permutation feature importance. The SHAP summary plot shown in Fig. 4 indicates that negative PE examination status is the most influential feature, substantially reducing the predicted probability of PE when present. Conversely, the presence of right-sided and left-sided PE contribute positively to PE prediction. Right ventricular strain features including (RV/LV ratio $\ge 1$ and RV/LV ratio $< 1$) exhibited directional effects, where higher values increased the predicted likelihood of PE consistent with established physiological mechanisms. Moderate contributions were observed for central PE and indeterminate cases, reflecting inter-patient variability. Lower ranked technical features such as (flow-artifact, qa-motion, qa-contrast), demonstrated minimal SHAP values and suggested limited influence on model predictions. In addition, The permutation feature importance in relation to change in the AUROC is shown in Fig. 5. The findings indicate that the most influential features for the proposed model are right-sided, left-sided and negative exam-for-pe. The feature rv-lv-ratio-gte-1 shows a moderate contribution to the model performance, whereas the remaining features such as central PE, chronic PE, and image quality-related variables demonstrate minimal impact. Overall, the explainability analysis confirms that embolus presence and laterality are key determinants of prediction.

Furthermore, an ablation study was conducted to investigate the contribution of each component in the proposed MPA-optimized-HSE model. As summarized in Table 6, removing SAINT from the ensemble led to a notable drop in performance (acc = 87.3%,F1-score = 85.56%), highlighting the critical role of the transformer as the strongest base learner. The configuration combining SAINT with MLP improved over the variant without SAINT (acc = 90.0%, F1-score = 86.0%), demonstrating the complementary contribution of the boosting learners (XGB, LGBM) when the transformer is present. Excluding the MPA optimization reduced performance compared to the full HSE model (acc = 90.6%, F1-score = 87.8%), confirming the effectiveness of adaptive weight optimization.

Additionally, a comparative analysis of different ensemble fusion strategies including hard voting, soft voting, manually weighted averaging, stacking with logistic regression (without MPA), and the proposed MPA-optimized-HSE with logistic regression was performed as shown in Table 5. The proposed model achieved the highest performance with an accuracy of (92.3%), precision (88.2%), recall (87.4%), and F1-score (89.5%). Hard voting yielded the lowest performance due to loses the probabilistic information, while soft voting improved results by averaging predicted probabilities. Manually weighted averaging provided further gains but remained inferior to the adaptive MPA-optimized stacking approach. Furthermore, stacking without MPA achieved competitive performance (F1-score = 87.8%), it was consistently outperformed by the fully optimized HSE. To further support the superiority of the proposed MPA-optimized-HSE model over baseline models, a Wilcoxon signed-rank test was conducted across the cross-validation folds for each performance metric. Table 8 summarizes the mean differences in performance, corresponding p-values, and statistical significance. The results indicate that MPA-optimized-HSE consistently outperforms all baseline models (SAINT, XGB, LGBM, and MLP) across Accuracy, Precision, Recall, and F1-score, with all differences being statistically significant (p< 0.05). These findings confirm that the observed improvements in model performance are robust and not due to random variation across folds.

**Table 4:** Performance and computational time comparison of different models on PE detection.

Model	Acc (%)	Pre (%)	Rec (%)	F1-score (%)	Training time/Fold (Avg.)
MLP	76.5	74.1	72.5	73.2	6.1 min
LGBM	78.3	75.5	74.3	72.6	2.4 min
XGB	85.7	82.1	80.8	81.6	3.6 min
SAINT	90.4	87.1	85.2	86.6	20.3 min
**MPA-optimized-HSE (Proposed)**	**92.3**	**88.2**	**87.4**	**89.5**	**41.7 min**

**Table 5:** Comparison of different stacking strategies of the proposed model.

Stacking Strategy	Acc (%)	Pre (%)	Rec (%)	F1-score (%)
Hard Voting	88.7	84.9	83.8	84.3
Soft Voting	89.6	85.8	84.9	85.3
Weighted Average	90.4	86.7	85.5	86.1
Stacking (LR, no MPA)	90.6	87.0	85.0	87.8
**MPA-optimized-HSE (LR + MPA) (Proposed)**	**92.3**	**88.2**	**87.4**	**89.5**

**Table 6:** Ablation study for the proposed framework.

Configuration	Acc (%)	Pre (%)	Rec (%)	F1-score (%)	Base Learners
HSE w/o SAINT	87.8	84.8	83.0	85.5	XGB + LGBM + MLP + LR + MPA
SAINT + MLP	90.0	86.5	84.5	86.0	SAINT + MLP + LR + MPA
HSE w/o MPA	90.6	87.0	85.0	87.8	SAINT + XGB + LGBM + MLP + LR
**MPA-optimized-HSE (Proposed)**	**92.3**	**88.2**	**87.4**	**89.5**	**SAINT + XGB + LGBM + MLP + LR + MPA**

**Table 7:** Comparison of prior studies on PE prediction using tabular clinical data.

Study	Data Type	Dataset availability	Method	Performance (Acc / AUROC)
Zhou et al.^24^	Tabular clinical (EHR)	Private	RF	Reported on private dataset
Ben Yehuda et al.^58^	Tabular clinical (admission data)	Private	Traditional ML	Reported on private dataset
Somani et al.^13^	Tabular clinical + ECG	Private	ML (EHR model)	Reported on private dataset
**MPA-optimized-HSE (Proposed)**	**Tabular clinical [RSNA-STR-PE]**	Public	**Hybrid Stacking Ensemble (SAINT + XGB + LGBM + MLP + MPA optimization)**	**Acc = 92.3%, AUROC = 0.91**

**Table 8:** Wilcoxon signed-rank test results comparing MPA-optimized-HSE with baseline models across different metrics.

Model Comparison	Metric	Mean Difference (%)	p-value	Significant (p<0.05)
MPA-optimized-HSE vs SAINT	Acc	1.9	0.031	Yes
MPA-optimized-HSE vs XGB	Acc	6.6	0.015	Yes
MPA-optimized-HSE vs LGBM	Acc	14.0	0.008	Yes
MPA-optimized-HSE vs MLP	Acc	15.8	0.007	Yes
MPA-optimized-HSE vs SAINT	Pre	1.1	0.034	Yes
MPA-optimized-HSE vs XGB	Pre	6.1	0.016	Yes
MPA-optimized-HSE vs LGBM	Pre	12.7	0.009	Yes
MPA-optimized-HSE vs MLP	Pre	14.1	0.007	Yes
MPA-optimized-HSE vs SAINT	Rec	2.2	0.029	Yes
MPA-optimized-HSE vs XGB	Rec	7.2	0.014	Yes
MPA-optimized-HSE vs LGBM	Rec	13.1	0.008	Yes
MPA-optimized-HSE vs MLP	Rec	14.9	0.006	Yes
MPA-optimized-HSE vs SAINT	F1-score	2.9	0.028	Yes
MPA-optimized-HSE vs XGB	F1-score	7.9	0.014	Yes
MPA-optimized-HSE vs LGBM	F1-score	16.9	0.005	Yes
MPA-optimized-HSE vs MLP	F1-score	16.3	0.006	Yes

## Discussion

In this research, we developed MPA-optimized-HSE model via MPA to detect PE using tabular clinical data of the RSNA-STR-PE dataset. The experimental results demonstrate that the proposed MPA-optimized HSE model achieves consistent and significant improvements over all baseline models. This improvement can be attributed to the complementary nature of the heterogeneous base learners, where tree-based models capture non-linear feature interactions, while transformer-based and neural network models learn complex feature dependencies. The ensemble strategy further enhances performance by integrating these diverse representations through adaptive weighting. Additionally, the use of MPA optimization contributes to improved generalization by jointly tuning both model hyperparameters and ensemble weights, resulting in more stable and robust predictions across cross-validation folds. This study is the first to integrate SAINT transformer, MLP, tree-based models within MPA optimized stacking framework for PE classification, providing a new approach that combines heterogeneous learners with metaheuristic optimization for improved predictive performance. The proposed model consistently outperformed all individual base models, achieving an accuracy of 92.3%, precision of 88.2%, recall of 87.4% and F1-score of 89.5%, highlighting the benefits of integrating heterogeneous learners and optimizing ensemble weights using a metaheuristic strategy. The ROC curve, correlation matrix, and permutation feature importance results (Figs. 2, 3, and 5) further confirmed the model’s strong discriminative power, demonstrating its ability to correctly classify PE-positive and PE-negative cases. Permutation feature importance and SHAP analysis consistently revealed that negative PE exams, right and left sided PE, and RV/LV ratios were the most influential features, consistent with known clinical indicators of PE severity. Weak correlations of imaging artifact-related features justified the use of an adaptive ensemble approach, allowing the model to weight more informative features appropriately.

Comparison with traditional ensemble strategies (hard voting, soft voting, manually weighted averaging, and stacking without MPA) emphasized the superiority of the proposed MPA-optimized-HSE. Adaptive weighting of base models was critical to achieving optimal performance, overcoming limitations of heuristic or non-adaptive methods. This suggests that adaptive weighting is crucial for effectively leveraging the strengths of different models, rather than relying on static or heuristic combination strategies.Also, the ablation study as obtained from Table 6 further confirmed the contributions of individual components. Removing SAINT from the ensemble reduced accuracy to 87.8% and F1-score to 85.5%, highlighting the critical role of the transformer. Excluding MPA optimization lowered performance to 90.6% accuracy and 87.8% F1-score, emphasizing the benefit of adaptive weight optimization. Combining SAINT with MLP achieved 90.0% accuracy and 86.0% F1-score, showing complementary contributions of boosting learners. Furthermore, Table 7 summarizes a comparison of prior studies using tabular clinical data for PE prediction alongside the proposed MPA-optimized-HSE model. To the best of our knowledge and based on the available literature, no previous study has utilized the publicly available RSNA-STR-PE dataset for tabular PE prediction. Previous works mainly reported results on private datasets, limiting direct quantitative comparison. Nevertheless, Table 7 highlights methodological differences and underscores the novelty, reproducibility, and standardized benchmarking enabled by our study.

Building upon the observed performance improvements presented in the Results section, the Wilcoxon signed-rank test was conducted to statistically validate the superiority of the proposed MPA-optimized-HSE model over baseline models. Across all performance metrics—Accuracy, Precision, Recall, and F1-score—the model consistently outperformed all baseline models (SAINT, XGB, LGBM, and MLP) with statistically significant differences (p< 0.05). These results confirm that the improvements observed are robust across cross-validation folds and highlight the effectiveness of integrating heterogeneous base learners with adaptive ensemble optimization. Overall, the findings support the conclusion that the proposed MPA-optimized-HSE framework provides a reliable, reproducible, and high-performing approach for PE prediction from tabular clinical data. By employing RSNA-STR-PE, our work provides the first reproducible evaluation of a metaheuristic-optimized hybrid stacking ensemble for PE prediction from tabular clinical data. Therefore, while prior studies provide valuable context, performance comparisons should be interpreted qualitatively rather than as direct head-to-head evaluation.

Despite these strengths, some limitations must be recognized. First, although the model was evaluated on RSNA-STR-PE dataset which improves generalizability, it remains retrospective and further validation on external datasets is warranted. Second, the model relies on features available in the tabular dataset and additional clinical or imaging features could further improve predictive performance. Future work will also explore alternative metaheuristic optimization strategies, and enhanced explainable AI techniques to support wider clinical adoption. In conclusion, the MPA-optimized-HSE technique provides a robust, interpretable, and high performing framework of PE diagnosis from tabular clinical data. The combination of strong predictive performance, feature interpretability via SHAP, and systematic optimization positions this approach as a promising tool for supporting clinical decision-making in real-world scenarios.

## Conclusion and future directions

Most previous studies on the RSNA-STR-PE dataset have focused on image-based ML approaches using CNNs or transformer architectures applied to CTPA scans. In contrast, our study is the first to utilize tabular clinical metadata with a metaheuristic-optimized hybrid stacking ensemble, highlighting a research gap that motivates our work. In this study, we proposed a MPA-optimized-HSE model for the prediction of PE using tabular clinical data. The proposed MPA-optimized-HSE integrates four complementary base models (SAINT transformer, MLP, XGB, and LGBM) and combines their probabilistic outputs through a logistic regression meta-learner. This study is the first to combine SAINT transformer with neural networks and tree-based models in MPA-optimized-HSE stacking framework for PE diagnosis, providing a new approach that leverages heterogeneous learners and metaheuristic optimization for enhanced predictive performance.

Experimental results on the RSNA-STR-PE dataset demonstrated that the optimized HSE achieved an accuracy of 92.3% with a precision of 88.2%, recall of 87.4%, and F1-score of 89.5%, outperforming all individual base models and alternative ensemble strategies. The incorporation of MPA effectively optimized the ensemble weights, enhancing model generalization and mitigating overfitting. Furthermore, SHAP-based interpretability analysis indicates that the model relies primarily on clinically relevant features such as embolus location and RV/LV ratio, whereas technical imaging artifacts contribute minimally. Overall, the proposed technique provides a robust, accurate, and interpretable framework for PE prediction, which has potential applicability in real-world clinical settings. The future directions includes extending the framework by integrating CTPA images with tabular clinical data to create a more comprehensive hybrid model potentially enhancing its predictive insights and performance. Additionally, alternative metaheuristic optimization strategies could be explored to further refine ensemble weight optimization and overall model performance. Furthermore, future deployment in real-time clinical decision support systems could further improve the model’s robustness and clinical utility.

## Data Availability

https://www.kaggle.com/c/rsna-str-pulmonary-embolism-detection/data.

## References

[1] Lanza, E., Ammirabile, A. & Francone, M. Meta-analysis of ai-based pulmonary embolism detection: How reliable are deep learning models?. *Comput. Biol. Med.* **193**, 110402 (2025). 40412084 10.1016/j.compbiomed.2025.110402

[2] Wu, H. et al. Pulmonet: a transmodal deep learning framework for automated pulmonary embolism diagnosis on non-contrast ct. *Biomed. Signal Process. Control* **113**, 108992 (2026).

[3] Cohen, A. et al. The number of vte events and associated morbidity and mortality. *Thromb Haemost* **98**, 756–764 (2007). 17938798 10.1160/TH07-03-0212

[4] Biret, C. B. et al. Advancing pulmonary embolism detection with integrated deep learning architectures. *J. Imaging Inform. Med.* **39**, 186–201 (2025). 40281216 10.1007/s10278-025-01506-6 PMC12921004

[5] Dua, R., Ronald Wallace, G., Chotso, T. & Francis Densil Raj, V. Classifying pulmonary embolism cases in chest ct scans using vgg16 and xgboost. In *Intelligent Communication Technologies and Virtual Mobile Networks: Proceedings of ICICV 2022*, 273–292 (Springer, 2022).

[6] Rivas, L. F. Clinical characterization of patients with venous thromboembolic disease in 2 reference centers in el salvador. *Blood* **142**, 5555 (2023).

[7] Sukumar, S. et al. Deep learning based pulmonary embolism detection using convolutional feature maps of ct pulmonary angiography images. *Procedia Comput. Sci.* **233**, 317–326 (2024).

[8] Abdelhamid, A., El-Ghamry, A., Abdelhay, E. H., Abo-Zahhad, M. M. & Moustafa, H.E.-D. Improved pulmonary embolism detection in ct pulmonary angiogram scans with hybrid vision transformers and deep learning techniques. *Sci. Rep.* **15**, 31443 (2025). 40858729 10.1038/s41598-025-16238-4 PMC12381140

[9] Guo, J. et al. Aanet: artery-aware network for pulmonary embolism detection in ctpa images. In *International conference on medical image computing and computer-assisted intervention*, 473–483 (Springer, 2022).

[10] Bass, A. R. et al. Clinical decision rules for pulmonary embolism in hospitalized patients: a systematic literature review and meta-analysis. *Thromb. Haemost.* **117**, 2176–2185 (2017). 29044295 10.1160/TH17-06-0395

[11] Belkouchi, Y. et al. Detection and quantification of pulmonary embolism with artificial intelligence: the sfr 2022 artificial intelligence data challenge. *Diagn. Interv. Imaging* **104**, 485–489 (2023). 37321875 10.1016/j.diii.2023.05.007

[12] Özkan, H., Osman, O., Şahin, S. & Boz, A. F. A novel method for pulmonary embolism detection in cta images. *Comput. Methods Progr. Biomed.* **113**, 757–766 (2014). 10.1016/j.cmpb.2013.12.014 24440133

[13] Somani, S. S. et al. Development of a machine learning model using electrocardiogram signals to improve acute pulmonary embolism screening. *Eur. Heart J.-Digit. Health* **3**, 56–66 (2022). 35355847 10.1093/ehjdh/ztab101 PMC8946569

[14] Kong, H. et al. Predicting the risk of pulmonary embolism in patients with tuberculosis using machine learning algorithms. *Eur. J. Med. Res.* **29**, 618 (2024). 39710777 10.1186/s40001-024-02218-3 PMC11664847

[15] Gutheil, J. & Donsa, K. Saintens: self-attention and intersample attention transformer for digital biomarker development using tabular healthcare real world data. In *dHealth 2022* 212–220 (IOS Press, 2022). 10.3233/SHTI220371 35592984

[16] Nanni, L., Brahnam, S., Loreggia, A. & Barcellona, L. Heterogeneous ensemble for medical data classification. *Analytics* **2**, 676–693 (2023).

[17] Colak, E. et al. The rsna pulmonary embolism ct dataset. *Radiol. Artif. Intell.* **3**, e200254 (2021). 33937862 10.1148/ryai.2021200254 PMC8043364

[18] Ma, X., Ferguson, E. C., Jiang, X., Savitz, S. I. & Shams, S. A multitask deep learning approach for pulmonary embolism detection and identification. *Sci. Rep.* **12**, 13087 (2022). 35906477 10.1038/s41598-022-16976-9 PMC9338063

[19] Shwartz-Ziv, R. & Armon, A. Tabular data: Deep learning is not all you need. *Inf. Fusion* **81**, 84–90 (2022).

[20] Sumon, M. S. I. et al. Cardiotabnet: a novel hybrid transformer model for heart disease prediction using tabular medical data. *Health Inf. Sci. Syst.* **13**, 44 (2025). 40703220 10.1007/s13755-025-00361-7 PMC12279641

[21] Tiwari, A. & Wadhawan, A. Ai in pulmonary embolism detection: a review of imaging datasets. In *2025 12th International Conference on Reliability, Infocom Technologies and Optimization (Trends and Future Directions) (ICRITO)*, 1–6 (IEEE, 2025).

[22] Eini, P., Eini, P., Serpoush, H., Rezayee, M. & Tremblay, J. Advancing mortality prediction in pulmonary embolism using machine learning algorithms–systematic review and meta-analysis. *Pulm. Circ.* **15**, e70166 (2025). 40985005 10.1002/pul2.70166 PMC12450341

[23] Liu, L. et al. Establishment of machine learning-based tool for early detection of pulmonary embolism. *Comput. Methods Programs Biomed.* **244**, 107977 (2024). 38113803 10.1016/j.cmpb.2023.107977

[24] Zhou, Q., Huang, R., Xiong, X., Liang, Z. & Zhang, W. Prediction of pulmonary embolism by an explainable machine learning approach in the real world. *Sci. Rep.* **15**, 835 (2025). 39755685 10.1038/s41598-024-75435-9 PMC11700180

[25] Silva, B. V., Marques, J., Menezes, M. N., Oliveira, A. L. & Pinto, F. J. Artificial intelligence-based diagnosis of acute pulmonary embolism: Development of a machine learning model using 12-lead electrocardiogram. *Rev. Port. Cardiol.* **42**, 643–651 (2023). 37001583 10.1016/j.repc.2023.03.016

[26] Nguyen, M. T. & Nguyen, A. Electrocardiogram based heartbeat detection using deep learning. *REV J. Electron. Commun.*10.21553/rev-jec.401 (2025).

[27] Saranya, K., Karthikeyan, U., Kumar, A. S., Salau, A. O. & Tin Tin, T. Densenet-abilstm: revolutionizing multiclass arrhythmia detection and classification using hybrid deep learning approach leveraging ppg signals. *Int. J. Comput. Intell. Syst.* **18**, 33 (2025).

[28] Tur, Y. et al. Mortality prediction of pulmonary embolism patients with deep learning and xgboost. In *2024 4th International Conference on Electrical, Computer, Communications and Mechatronics Engineering (ICECCME)*, 01–06 (IEEE, 2024).

[29] Mulam, H., Chikati, V. R. & Kulkarni, A. Enhancing pulmonary embolism segmentation through optimized swinunet with resnet 152. *J. Inst. Eng. India Ser. B* **106**, 1691–1699 (2025).

[30] Singh, G. et al. Comparing efficiency of an attention-based deep learning network with contemporary radiological workflow for pulmonary embolism detection on ctpa: A retrospective study. *Eur. J. Radiol. Open* **14**, 100657 (2025). 40469717 10.1016/j.ejro.2025.100657 PMC12136827

[31] Cahan, N. et al. Multimodal fusion models for pulmonary embolism mortality prediction. *Sci. Rep.* **13**, 7544 (2023). 37160926 10.1038/s41598-023-34303-8 PMC10170065

[32] Kumar, A. S. & Rekha, R. A dense network approach with gaussian optimizer for cardiovascular disease prediction. *New Gener. Comput.* **41**, 859–878 (2023).

[33] Faramarzi, A., Heidarinejad, M., Mirjalili, S. & Gandomi, A. H. Marine predators algorithm: A nature-inspired metaheuristic. *Expert Syst. Appl.* **152**, 113377 (2020).

[34] Rezk, S. S. & Selim, K. S. Metaheuristic-based ensemble learning: an extensive review of methods and applications. *Neural Comput. Appl.* **36**, 17931–17959 (2024).

[35] Lian, Z., Wei, X.-N. & Chai, D. Machine learning-based prediction of pulmonary embolism prognosis using nutritional and inflammatory indices. *Clin. Appl. Thromb./Hemost.* **30**, 10760296241300484 (2024). 39552298 10.1177/10760296241300484 PMC11571247

[36] Mesinovic, M. et al. At-admission prediction of mortality and pulmonary embolism in an international cohort of hospitalised patients with covid-19 using statistical and machine learning methods. *Sci. Rep.* **14**, 16387 (2024). 39013928 10.1038/s41598-024-63212-7 PMC11252333

[37] Wang, G. et al. Machine learning-based models for predicting mortality and acute kidney injury in critical pulmonary embolism. *BMC Cardiovasc. Disord.* **23**, 385 (2023). 37533004 10.1186/s12872-023-03363-z PMC10399014

[38] Ma, R. et al. Machine learning in the prediction of venous thromboembolism: Systematic review and meta-analysis. *J. Med. Internet Res.* **27**, e77339 (2025). 41433036 10.2196/77339 PMC12724482

[39] Kim, J. S. et al. Machine learning-based prediction of pulmonary embolism to reduce unnecessary computed tomography scans in gastrointestinal cancer patients: a retrospective multicenter study. *Sci. Rep.* **14**, 25359 (2024). 39455658 10.1038/s41598-024-75977-y PMC11511972

[40] Sezer, M., Turtay, M. G., Yıldırım, H., Yaşar, Ş & Küçükakçalı, Z. Use of artificial intelligence in pulmonary embolism prediction. *Eurasian J. Emerg. Med.* **24**, 300–308 (2025).

[41] RSNA. Rsna pulmonary embolism detection challenge. https://www.rsna.org/Research/PE-Challenge (2020).

[42] Kelleher, J. D., Mac Namee, B. & D’arcy, A. *Fundamentals of machine learning for predictive data analytics: algorithms, worked examples, and case studies* (MIT press, 2020).

[43] Hastie, T., Tibshirani, R., Friedman, J. & Franklin, J. The elements of statistical learning: data mining, inference and prediction. *Math. Intell.* **27**, 83–85 (2005).

[44] Goodfellow, I. *Deep Learning* (MIT press, 2016).

[45] Bishop, C. M. & Nasrabadi, N. M. *Pattern Recognition and Machine Learning* Vol. 4 (Springer, 2006).

[46] Tian, J. et al. High-performance fault classification based on feature importance ranking-xgboost approach with feature selection of redundant sensor data. *Current Chin. Sci.* **2**, 243–251 (2022).

[47] Li, J. et al. Application of xgboost algorithm in the optimization of pollutant concentration. *Atmos. Res.* **276**, 106238 (2022).

[48] Noviandy, T. R., Idroes, G. M. & Hardi, I. An interpretable machine learning strategy for antimalarial drug discovery with lightgbm and shap. *J. Future Artif. Intell. Technol.* **1**, 84–95 (2024).

[49] Somepalli, G., Goldblum, M., Schwarzschild, A., Bruss, C. B. & Goldstein, T. Saint: Improved neural networks for tabular data via row attention and contrastive pre-training. arXiv preprint arXiv:2106.01342 (2021).

[50] Vaswani, A. et al. Attention is all you need. *Advances in neural information processing systems* **30** (2017).

[51] Ba, J. L., Kiros, J. R. & Hinton, G. E. Layer normalization. arXiv preprint arXiv:1607.06450 (2016).

[52] Nair, V. & Hinton, G. E. Rectified linear units improve restricted Boltzmann machines. In *Proceedings of the 27th international conference on machine learning (ICML-10)*, 807–814 (2010).

[53] Al-Betar, M. A. et al. Marine predators algorithm: A review. *Arch. Comput. Methods Eng.* **30**, 3405–3435 (2023). 37260911 10.1007/s11831-023-09912-1 PMC10115392

[54] Mugemanyi, S. et al. Marine predators algorithm: A comprehensive review. *Mach. Learn. Appl.* **12**, 100471 (2023).

[55] Zhang, Z. Improved adam optimizer for deep neural networks. In *2018 IEEE/ACM 26th international symposium on quality of service (IWQoS)*, 1–2 (IEEE, 2018).

[56] Abdelhaliem, I., Dixon, J., Abdelhamid, A., Saleh, G. A. & Khalifa, F. A multimodal adaptive inter-region attention-guided network for brain tumor classification. *IEEE Access* **13**, 187964–187975 (2025). 41357810 10.1109/access.2025.3627777 PMC12680093

[57] Chicco, D. & Jurman, G. The advantages of the Matthews correlation coefficient (mcc) over f1 score and accuracy in binary classification evaluation. *BMC Genom.* **21**, 6 (2020). 10.1186/s12864-019-6413-7 PMC6941312 31898477

[58] Ben Yehuda, O., Itelman, E., Vaisman, A., Segal, G. & Lerner, B. Early detection of pulmonary embolism in a general patient population immediately upon hospital admission using machine learning to identify new, unidentified risk factors: model development study. *J. Med. Internet Res.* **26**, e48595 (2024). 39079116 10.2196/48595 PMC11322683

